# Machine learning to predict bacteriologic confirmation of *Mycobacterium tuberculosis* in infants and very young children

**DOI:** 10.1371/journal.pdig.0000249

**Published:** 2023-05-17

**Authors:** Jonathan P. Smith, Kyle Milligan, Kimberly D. McCarthy, Walter Mchembere, Elisha Okeyo, Susan K. Musau, Albert Okumu, Rinn Song, Eleanor S. Click, Kevin P. Cain

**Affiliations:** 1 Department of Health Policy and Management, Yale School of Public Health, New Haven, Connecticut, United States of America; 2 Division of Global HIV and Tuberculosis, Centers for Disease Control and Prevention, Atlanta, Georgia, United States of America; 3 Peraton, Atlanta, Georgia, United States of America; 4 Center for Global Health Research, Kenya Medical Research Institute, Kisumu, Kenya; 5 Oxford Vaccine Group, Department of Paediatrics, University of Oxford, Oxford, United Kingdom; 6 Department of Pediatrics, Harvard Medical School, Boston, Massachusetts, United States of America; University of Toronto, CANADA

## Abstract

Diagnosis of tuberculosis (TB) among young children (<5 years) is challenging due to the paucibacillary nature of clinical disease and clinical similarities to other childhood diseases. We used machine learning to develop accurate prediction models of microbial confirmation with simply defined and easily obtainable clinical, demographic, and radiologic factors. We evaluated eleven supervised machine learning models (using stepwise regression, regularized regression, decision tree, and support vector machine approaches) to predict microbial confirmation in young children (<5 years) using samples from invasive (reference-standard) or noninvasive procedure. Models were trained and tested using data from a large prospective cohort of young children with symptoms suggestive of TB in Kenya. Model performance was evaluated using areas under the receiver operating curve (AUROC) and precision-recall curve (AUPRC), accuracy metrics. (i.e., sensitivity, specificity), F-beta scores, Cohen’s Kappa, and Matthew’s Correlation Coefficient. Among 262 included children, 29 (11%) were microbially confirmed using any sampling technique. Models were accurate at predicting microbial confirmation in samples obtained from invasive procedures (AUROC range: 0.84–0.90) and from noninvasive procedures (AUROC range: 0.83–0.89). History of household contact with a confirmed case of TB, immunological evidence of TB infection, and a chest x-ray consistent with TB disease were consistently influential across models. Our results suggest machine learning can accurately predict microbial confirmation of *M*. *tuberculosis* in young children using simply defined features and increase the bacteriologic yield in diagnostic cohorts. These findings may facilitate clinical decision making and guide clinical research into novel biomarkers of TB disease in young children.

## Author summary

Pediatric tuberculosis (TB) is a leading cause of child mortality, yet diagnosis of TB in children is one of the leading challenges in pediatric diagnostic medicine. Many symptoms of TB are similar to other childhood illnesses, therefore a positive laboratory specimen is the only way to confirm TB disease. Unfortunately, children naturally produce less bacteria than adults, and laboratory confirmed TB disease in children is rare. This diagnostic challenge contributes to delays in treatment initiation and unacceptable child mortality: 96% of TB-related child deaths are among children not receiving treatment. In thus study, we use data from a cohort of children with TB-like symptoms to develop machine learning algorithms that can predict if a child is likely to produce a positive specimen with a high degree of accuracy. These findings could facilitate clinical decision making and support treatment initiation earlier in the clinical course of disease.

## Introduction

Tuberculosis (TB), an airborne infectious disease caused by *Mycobacterium tuberculosis*, remains a major global cause of morbidity and mortality among children under the age of 15. An estimated one million children fall ill with TB annually and over a quarter-million children die from TB disease [1]. Mortality among pediatric TB cases is most profound among infants and young children under 5 years of age [1]. Young children have an estimated mortality rate of over nine times that in older children and adolescents (5–15 years) and account for almost 80% of all TB deaths under 15 years old [2]. The vast majority of TB mortality among infants and young children (96%) is among children not receiving anti-TB treatment [2]. Such data highlight the potential for drastic reductions in TB mortality through strategies to improve diagnosis and treatment initiation among pediatric TB patients.

Microbial confirmation of TB disease remains among the most pressing challenges facing clinicians and researchers seeking to accurately diagnose TB and initiate treatment in young children. Pediatric TB is paucibacillary by nature and the primary specimen used to confirm TB disease in adults, expectorated sputum, is not feasible to collect from young children. The most accurate reference standards for specimen collection in young children, gastric aspirate and induced sputum, require highly invasive procedures that often cause significant physical and mental discomfort to the child and family [3]. Unfortunately, despite ideal scenarios these invasive procedures remain suboptimal, with a diagnostic yield of only 25–50 percent in high-resource settings [3,4]. Recent work has investigated a collection of alternative specimen collection procedures using minimally- or noninvasive procedures, such as oral swabs, nasopharyngeal aspirate, urine, or stool samples [5–8]. While more comfortable and feasible in limited-resource settings, these combinations typically result in similar or lower bacteriologic yields [5–8].

In the absence of microbial confirmation, clinical diagnosis (diagnosis using only symptoms and patient history without a lab-confirmed specimen) is the *de facto* method to identify pediatric TB cases. Clinical diagnoses are complicated by nonspecific symptoms that often overlap with other common childhood infections, such as cough and fever. As a consequence of these diagnostic limitations in young children, it is widely acknowledged that the majority of pediatric TB patients remain under- or undiagnosed and untreated [2].

Accurate prediction of microbially confirmed TB cases among young children suspected of TB disease and the identification of factors that contribute to a positive result would allow for targeted strategies in both clinical decision-making and future diagnostic research efforts. Using easily obtainable clinical, demographic, and radiological data from a large prospective cohort of young children with symptoms concerning for TB disease, we designed and evaluated eleven machine-learning based classification models to predict microbially confirmed TB diagnoses in young children. The primary aim of this study is to determine if machine learning methods could accurately predict microbial confirmation from suspected pediatric TB patients using samples obtained from both invasive (reference standard) or noninvasive specimen collection procedures. We compared multiple model metrics to examine and compare performance across machine learning approaches and examined the influence of clinical and demographic factors in the model selection process.

## Materials and methods

### Study enrollment

The *M’toto* study (“*little child*” in Swahili) is a prospective, diagnostic cross-sectional study conducted between October 2013 and August 2015 at inpatient and outpatient clinics serving urban, peri-urban, and rural communities in the greater Kisumu County, Kenya area. Full study details and enrollment criteria are described in detail elsewhere [5]. Briefly, all infants and young children (<5 years) presenting with clinical signs and symptoms of TB were screened for study inclusion. Enrolled children presented with cough, fever, moderate to severe malnutrition, and visible cervical lymph node mass measuring >1 cm x 1cm or parenchymal abnormality on chest x-ray. Children were excluded if they were on anti-TB treatment or TB preventative therapy in the last year or 6 months, respectively.

The purpose of the *M’toto* study was to identify combinations of both invasive and minimally invasive bacteriologic specimen collection procedures that produced the highest yield of bacteriologic confirmed TB diagnosis. Clinical study staff collected a panel of up to eight specimen types, including two samples each of the current invasive reference standard procedures (gastric aspirate (GA) and induced sputum (IS)), as well as samples from the minimally invasive procedures of nasopharyngeal aspirate (NPA), stool, string test (ST), and urine. Two samples of cervical lymph node fine-needle aspirate (FNA) were taken if indicated, and a single sample of blood was taken. Samples were collected within three days of study enrollment. The panel was tested for microbial confirmation with both the PCR-based Xpert MTB/RIF (Xpert) and mycobacteria growth indicator tube (MGIT).

This study was reported in accordance with the Transparent Reporting of a Multivariable Prediction Model for Individual Prognosis or Diagnosis (TRIPOD) statement.

### Ethical approval

The study was approved by the institutional review boards (IRBs) of the U.S. Centers for Disease Control and Prevention (CDC), the Kenya Medical Research Institute, and the Jaramogi Oginga Odinga Teaching and Referral Hospital. Harvard Medical School relied on the review and oversight of the CDC IRB. Written informed consent was obtained by parents or legal guardians of participants.

### Model outcomes, predictors, and clinical definitions

We developed machine learning models to predict the primary outcome of microbial confirmation using (1) only specimens obtained from the current reference-standard, invasive procedures (GA or IS; “invasive”) or (2) specimens obtained using only noninvasive procedures (NPA, stool, ST, urine, blood, and FNA; “noninvasive”).

We included predictor variables obtained from easily identifiable clinical and demographic factors at the first clinical encounter from suspected pediatric TB patients. For the purposes of this analysis, we intentionally used simplified categorical definitions for each factor that are more applicable to limited-resource setting. These included: 1) age at enrollment (categorized as <1 year, 1–2 years, 2–3 years, 3–4 years, and 4–5 years), 2) biological sex (male or female), 3) persistent unexplained cough (“cough,” dichotomous; ≥ 4 weeks at encounter despite non-TB antibiotic treatment), 4) persistent unexplained fever (“fever,” dichotomous; ≥ 1 week despite non-TB antibiotic/antimalarials), 5) persistent unexplained lethargy (“lethargy,” dichotomous; ≥ 30 days despite antibiotics/antimalarials for five or more days), 6) malnutrition (categorized as “none,” “moderate,” or “severe”), 7) immunological evidence of TB infection (dichotomous; positive tuberculin skin test (TST) or interferon-gamma release assay (IGRA)), 8) chest x-ray (CXR) results consistent with TB disease (dichotomous), 9) history of household *M*. *tuberculosis* exposure (“history of exposure”; dichotomous), and 10) HIV status (dichotomous; HIV positive/HIV negative). Malnutrition categories were defined using standardized weight-for-age (WFA) z-scores as a proxy, calculated by World Health Organization’s (WHO’s) method for reporting on anthropometric indicators in children under 5 years old [9]. “Severe” malnutrition was defined as a z-score of ≤ -2.0, "moderate” malnutrition as a z-score between -2.0 and -1.0, and “none” as a z-score of > -1.0. History of household exposure was defined as a household contact with bacteriologically confirmed case within 12 months of enrollment [10]. Chest radiographs (CXR) were defined as consistent with TB disease after retrospective examination of digital films by at least two expert readers, with any disagreements resolved by a third reader. CXR results were dichotomized as either consistent with TB disease or not consistent with TB disease, the latter including both normal readings and abnormal readings not considered to be consistent with TB disease. For dichotomous predictors, the reference was considered the absence of the predictor; we used ordinal encoding for the polytomous predictors of age and malnutrition, with age less than 1 years and no malnutrition as the reference, respectively.

### Machine learning model selection and validation

We used four supervised machine learning frameworks to develop predictive models of microbial confirmation in very young children: stepwise regression, regularization, decision tree, and support vector machine (SVM) methods [11,12]. We consider machine learning models as those for which predictor variables are evaluated and determined for model inclusion without human decision making. This approach contrasts with classic *a priori* covariate selection in hypothesis-driven epidemiologic models traditionally used in pediatric diagnostic studies.

We first evaluated stepwise regression models with either stepwise forward selection, backwards elimination, or bidirectional elimination (i.e., both stepwise forward selection and backward elimination). These approaches solely prioritize the model with the lowest Akaike Information Criterion (AIC) for parameter selection. We then developed regression models using Ridge, Least Absolute Shrinkage and Selection Operator (LASSO), or Elastic Net regularization. Regularization methods use a penalty term and a free parameter, *λ*, to limit the size of the predictor coefficient (i.e., the *β′s*) in the logistic model [13]. In Ridge regularization, the penalty term reduces the coefficients that contribute most to the error (“shrinkage”); in LASSO regularization, the penalty term fully eliminates inconsequential coefficients from the model (sets *β*’s to zero). Elastic Net regularization combines the penalties of Ridge and LASSO by introducing an additional free parameter, *α* (where 0 ≤ *α* ≤ 1), to the penalty term such that the penalty falls between ridge and LASSO. Optimal *λ* and *α* values were selected using cross validation.

We evaluated two decision tree classification techniques, Random Forest and Gradient Boosted Trees. Random Forest classification algorithms use a large number of uncorrelated individual decision trees, each with a randomly selected subsets of covariates [14]. Through randomization, some trees will isolate more important covariates and thus the ensemble model is more accurate than any single decision tree. In this analysis, we used the classical choice of selecting 1000 trees, with the optimal number of covariates for each tree determined by cross validation. In contrast to Random Forest, Gradient Boosting builds decision trees one at a time, with each subsequent tree learning from the error in the previous to find the optimal model [13,15].

Lastly, we developed three support vector machine (SVM) models. SVM is a robust prediction technique that classifies data in a *j*-dimensional space (where *j* is the number of explanatory/predictor variables) and determines a decision boundary using a hyperplane (i.e., observations falling on one side of the boundary have the outcome of interest, and on the other do not) [16]. SVMs use a set of mathematical functions known as a kernel to transform the data into the required multidimensional format. By doing so, SVMs seek to optimize the margin between classes of data points thus ensuring the model is robust when applied to new datasets. In this analysis, we use three kernel selections to represent different abilities of the model to separate the data in a multidimensional space: linear, polynomial, and Radial Basis Function (RBF).

**Fig 1:**
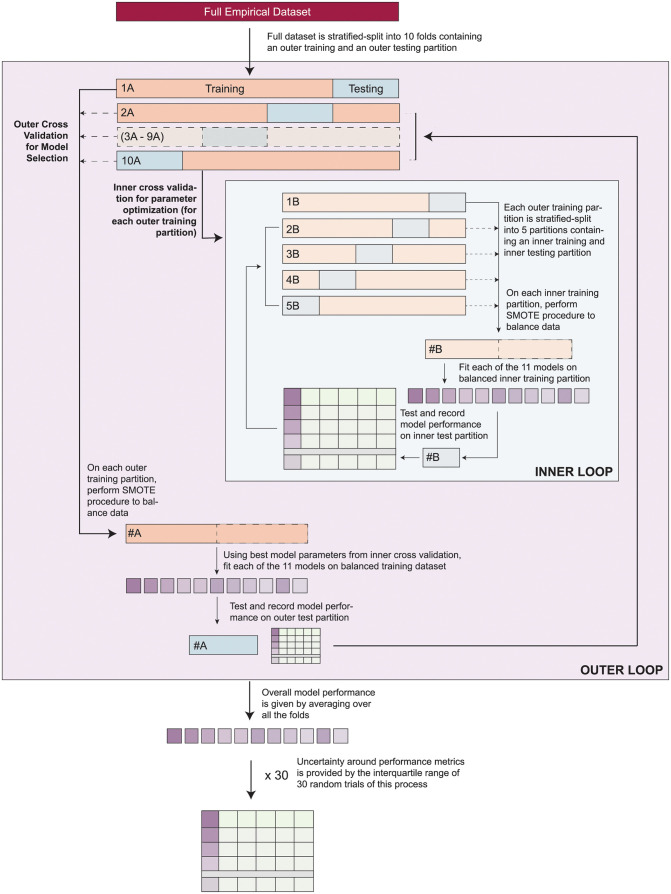
Modelling approach and evaluation. We performed nested cross validation to optimize parameters and examine model selection.

We used nested *k*-fold cross validation for model selection and hyperparameter tuning for all models, with k = 10 for the outer loop and k = 5 for the inner loop (Fig 1). Briefly, we first split the full dataset into 10 outer training (70 percent) and testing (30 percent) datasets using stratified random sampling to preserve the distribution of outcomes and predictors in both the testing and training datasets. We subsequently split each of the 10 outer training datasets into five inner training and testing datasets (70/30 split, respectively, also using stratified random sampling). We fit candidate models on the inner training data and evaluated performance on the inner testing data to optimize hyperparameters for each model. Using the best performing hyperparameters from the inner cross validation loop, we fit each model to the outer training dataset and calculated performance measures on the outer testing for all 10 partitions. Overall model performance for the total nested cross validation procedure was given by averaging metrics over all the folds. To quantify the degree of uncertainty around estimates of performance and examine model instability, we report the median and interquartile range (IQR) of accuracy measures from 30 repeated nested *k-*fold validation procedures. Results were averaged over multiple random seeds.

Microbial confirmation in young children is a rare event, and thus the cohort data are heavily imbalanced and may artificially increase the accuracy of naïve models. To address this concern, training data were balanced using the Synthetic Minority Oversampling Technique (SMOTE) [17]. SMOTE was performed on each training dataset within each fold of the inner and outer loops before model fitting. Overall model performance for was given by averaging metrics over all folds in the outer loop. Analyses were completed with participants for whom data on all predictor variables was available. For SVM models, Shapley Additive Explanations (SHAP) values were calculated to examine feature importance [18].

### Model performance

We primarily evaluated predictive performance of the models using the areas under the receiver operating characteristics curve (AUROC), which calculates the performance of a model across all possible classification thresholds. We defined high accuracy as an AUROC above 0.85, moderate accuracy as an AUROC between 0.75 and 0.85, and poor accuracy as an AUROC below 0.75 [19]. We also calculated the area under the precision-recall curve (AUPRC), a commonly used metric in imbalanced data to measure the model’s ability to identify rare events [20]. As the AUPRC is a function of the proportion of positives, we did not define performance categories *a priori*. We then calculated Youden’s *j* index to identify the point on the receiver operating curve that is farthest from random chance (i.e., the optimized cut point) [21]. Using these optimized thresholds, we further explore individual model accuracy calculating misclassification error (total number of incorrect predictions divided by total predictions), sensitivity, specificity, positive predictive value (PPV), and negative predictive value (NPV). We then calculated the F1 score as a comparative measure between algorithms, which is the harmonic mean of the sensitivity and PPV. As the F1 score weights sensitivity and PPV equally, we extended the F-measure to place more weight either on sensitivity (F2 score; more important to minimize false negatives) or PPV (F0.5 score; more important to minimize false positives). Lastly, we calculated Cohen’s Kappa and Matthew’s Correlation Coefficient (MCC), both of which assess the agreement between the predicted and actual values [22]. All analysis was performed in using R statistical software (version 4.1.2) [23]. All models, full model code, and data to recreate this analysis can be found on the GitHub repository, https://github.com/jpsmithuga/ML_Mtb_peds.

## Results

A total of 300 children under 5 years old suspected of having clinical TB disease were enrolled, among which 32 (11%) had microbial confirmation by at least one specimen from any collection technique. Complete case information suitable for analysis was available for 262 (87%) children, of whom 29 (11%) were bacteriologically confirmed from any specimen: 22 (76%) with at least one sample from both invasive and noninvasive sample collection procedures, 3 (10%) with samples from invasive procedures only, and 4 (14%) with samples from noninvasive procedures only. Among those included in the analysis, the median age was 739 days (2.0 years) with an interquartile range (IQR) of 380.5 to 1326.0 days (1.0 to 3.6 years); 131 (50%) were female and 65 (25%) were HIV positive (Table 1).

**Table 1:** Characteristics of study participants, by specimen collection technique, n (%).

		Any Specimen		Invasive Specimens Only		Noninvasive Specimens Only	
Characteristic	Total (n = 262)	Microbially confirmed (n = 29)	Not microbially confirmed (n = 233)	Microbially confirmed (n = 25)	Not microbially confirmed (n = 237)	Microbially confirmed (n = 26)	Not microbially confirmed (n = 236)
Age							
0–1 Years	65 (25%)	8 (28%)	57 (24%)	7 (28%)	58 (24%)	7 (27%)	58 (25%)
1–2 Years	65 (25%)	9 (31%)	56 (24%)	8 (32%)	57 (24%)	7 (27%)	58 (25%)
2–3 Years	38 (15%)	3 (10%)	35 (15%)	3 (12%)	35 (15%)	3 (12%)	35 (15%)
3–4 Years	55 (21%)	6 (21%)	49 (21%)	4 (16%)	51 (22%)	6 (23%)	49 (21%)
4–5 Years	39 (15%)	3 (10%)	36 (15%)	3 (12%)	36 (15%)	3 (12%)	36 (15%)
Female Sex	130 (50%)	14 (48%)	116 (50%)	12 (48%)	118 (50%)	13 (50%)	117 (50%)
HIV Positive	65 (25%)	6 (21%)	59 (25%)	5 (20%)	60 (25%)	6 (23%)	59 (25%)
Prolonged Cough	213 (81%)	24 (83%)	189 (81%)	20 (80%)	193 (81%)	22 (85%)	191 (81%)
Prolonged Fever	118 (45%)	16 (55%)	102 (44%)	14 (56%)	104 (44%)	16 (62%)	102 (43%)
Prolonged Lethargy	28 (11%)	7 (24%)	21 (9%)	7 (28%)	21 (9%)	7 (27%)	21 (9%)
Malnutrition							
Severe	58 (22%)	5 (17%)	53 (23%)	4 (16%)	54 (23%)	4 (15%)	54 (23%)
Moderate	118 (45%)	14 (48%)	104 (45%)	12 (48%)	106 (45%)	12 (46%)	106 (45%)
None	86 (33%)	10 (34%)	76 (33%)	9 (36%)	77 (32%)	10 (38%)	76 (32%)
CXR consistent with TB	44 (17%)	13 (45%)	31 (13%)	11 (44%)	33 (14%)	13 (50%)	31 (13%)
Positive TST/IGRA	54 (21%)	19 (66%)	35 (15%)	18 (72%)	36 (15%)	18 (69%)	36 (15%)
History of TB Exposure	79 (30%)	25 (86%)	54 (23%)	22 (88%)	57 (24%)	22 (85%)	57 (24%)

Microbial confirmation determined by positive Xpert or mycobacteria growth indicator tube (MGIT). TB, tuberculosis; CXR, chest radiograph; TST, tuberculin skin test; IGRA, interferon-gamma release assay. Specimens from invasive procedures include gastric aspirate and induced sputum. Specimens from noninvasive procedures include nasopharyngeal aspirate, stool, string test, cervical lymph node fine-needle aspirate, urine, and blood.

### Model performance

**Fig 2:**
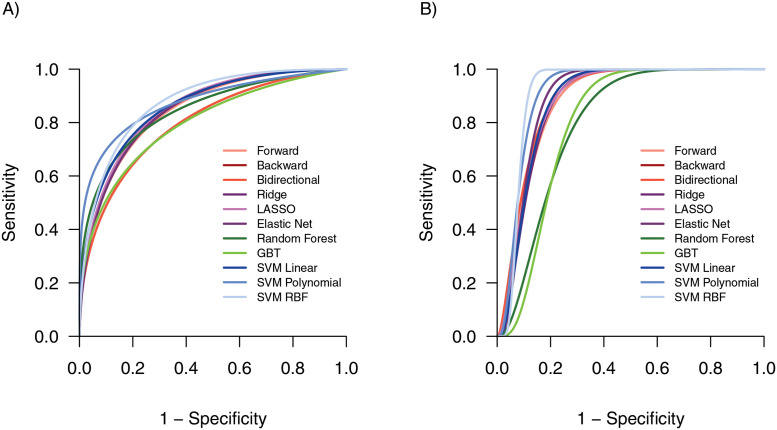
Comparison of receiver-operator curves (ROC) for model types, by specimen collection technique. ROC curves for models predicting the outcomes of: (A) Microbial confirmation using invasive specimens only (gastric aspirate and induced sputum), and (B) microbial confirmation using noninvasive specimens only (nasopharyngeal aspirate, stool, string test, cervical lymph node fine-needle aspirate, urine, and blood). GBT, Gradient Boosted Tree; SVM, Support Vector Machines; RBF, radial basis function.

For samples obtained from reference-standard invasive procedures (GA and IS), the performance for all models was classified as highly or moderately accurate in predicting microbial confirmation, with a median AUROC of 0.89 (range: 0.84–0.90; Fig 2, Table 2). However, when considering the comprehensive range of metrics used to examine model performance, there was substantial heterogeneity between models (Table 2), particularly those which prioritize the correct classification of positive samples (AUPRC, sensitivity, PPV, F2). The median AUPRC estimate was 0.46 (range: 0.39–0.52), suggesting a substantial increase in predictive ability over baseline (~0.10 for a random estimator in these data). Among modeling techniques from specimens using invasive procedures, tree-based models demonstrated the highest overall performance by measure of AUROC and AUPRC, however SVM models demonstrated lower overall misclassification error in predicting microbial confirmation (Table 2). Despite a lower sensitivity (0.86, IQR: 0.86, 1.00), the SVM Polynomial model consistently demonstrated the lowest overall misclassification (0.14, IQR: 0.10, 0.19) and the highest specificity (0.85, IQR: 0.79, 0.89), PPV (0.38, IQR: 0.32, 0.48), F1 score (0.53, IQR: 0.48, 0.52), F2 score (0.43, IQR: 0.37, 0.53), Cohen’s Kappa (0.45, IQR: 0.40, 0.56), and MCC (0.53, IQR: 0.50, 0.60).

**Table 2:** Comparison of machine learning models to predict microbial confirmation using invasive sampling procedures.

Model Type		Performance		Accuracy Metrics at Youden’s *j* index					Weighted Averages			Correlation Metrics	
		AUROC	AUPRC	Misclass.	Sensitivity	Specificity	PPV	NPV	F1 Score	F2 Score	F0.5 Score	Cohen’s Kappa	MCC
Stepwise Regression	Forward Selection	0.89 (0.86, 0.91)	0.46 (0.31, 0.52)	0.19 (0.14, 0.26)	**1.00 (0.86, 1.00)**	0.80 (0.73, 0.86)	0.32 (0.26, 0.38)	1.00 (0.98, 1.00)	0.48 (0.40, 0.54)	0.37 (0.30, 0.43)	0.68 (0.60, 0.72)	0.40 (0.30, 0.46)	0.49 (0.39, 0.54)
	Backward Elimination	0.89 (0.86, 0.91)	0.46 (0.31, 0.52)	0.19 (0.14, 0.26)	**1.00 (0.86, 1.00)**	0.80 (0.73, 0.86)	0.32 (0.26, 0.38)	1.00 (0.98, 1.00)	0.48 (0.40, 0.54)	0.37 (0.30, 0.43)	0.68 (0.60, 0.72)	0.40 (0.30, 0.46)	0.49 (0.39, 0.54)
	Bidirectional	0.89 (0.88, 0.92)	0.45 (0.40, 0.57)	0.17 (0.13, 0.26)	0.86 (0.86, 1.00)	0.82 (0.72, 0.87)	0.35 (0.26, 0.40)	0.99 (0.98, 1.00)	0.51 (0.41, 0.56)	0.40 (0.31, 0.45)	0.69 (0.64, 0.74)	0.43 (0.32, 0.49)	0.50 (0.43, 0.56)
Regularization	LASSO	0.89 (0.87, 0.92)	0.49 (0.35, 0.53)	0.18 (0.14, 0.26)	**1.00 (0.86, 1.00)**	0.81 (0.72, 0.85)	0.33 (0.25, 0.39)	1.00 (0.98, 1.00)	0.49 (0.40, 0.56)	0.38 (0.29, 0.43)	0.66 (0.62, 0.73)	0.41 (0.30, 0.48)	0.48 (0.42, 0.56)
	Ridge	0.89 (0.88, 0.92)	0.47 (0.39, 0.56)	0.17 (0.14, 0.22)	**1.00 (0.86, 1.00)**	0.82 (0.76, 0.86)	0.33 (0.29, 0.39)	1.00 (0.98, 1.00)	0.50 (0.44, 0.55)	0.38 (0.33, 0.44)	0.69 (0.64, 0.74)	0.42 (0.35, 0.48)	0.51 (0.43, 0.55)
	Elastic Net	0.88 (0.85, 0.90)	0.40 (0.27, 0.51)	0.21 (0.15, 0.26)	**1.00 (0.86, 1.00)**	0.78 (0.73, 0.83)	0.29 (0.24, 0.36)	1.00 (0.98, 1.00)	0.44 (0.39, 0.52)	0.34 (0.28, 0.41)	0.64 (0.59, 0.68)	0.36 (0.29, 0.44)	0.45 (0.38, 0.50)
Decision Tree	Random Forest	**0.90 (0.88, 0.93)**	0.46 (0.40, 0.57)	0.21 (0.14, 0.24)	**1.00 (1.00, 1.00)**	0.78 (0.73, 0.86)	0.29 (0.27, 0.38)	**1.00 (1.00, 1.00)**	0.46 (0.43, 0.54)	0.34 (0.32, 0.43)	0.67 (0.64, 0.75)	0.37 (0.33, 0.47)	0.47 (0.44, 0.55)
	Gradient Boosted Tree	**0.90 (0.87, 0.92)**	**0.52 (0.42, 0.60)**	0.17 (0.13, 0.22)	**1.00 (0.86, 1.00)**	0.82 (0.76, 0.86)	0.35 (0.29, 0.41)	1.00 (0.98, 1.00)	0.52 (0.44, 0.57)	0.40 (0.33, 0.46)	**0.72 (0.64, 0.73)**	0.44 (0.35, 0.51)	0.53 (0.45, 0.55)
Support Vector Machines	SVM Linear	**0.90 (0.87, 0.92)**	0.48 (0.36, 0.60)	0.19 (0.13, 0.25)	**1.00 (0.86, 1.00)**	0.79 (0.73, 0.87)	0.30 (0.26, 0.41)	1.00 (0.98, 1.00)	0.46 (0.41, 0.57)	0.35 (0.31, 0.46)	0.68 (0.63, 0.73)	0.38 (0.31, 0.51)	0.49 (0.43, 0.58)
	SVM Polynomial	0.89 (0.87, 0.93)	0.46 (0.37, 0.57)	**0.14 (0.10, 0.19)**	0.86 (0.86, 1.00)	**0.85 (0.79, 0.89)**	**0.38 (0.32, 0.48)**	0.99 (0.98, 1.00)	**0.53 (0.48, 0.62)**	**0.43 (0.37, 0.53)**	0.70 (0.69, 0.75)	**0.45 (0.40, 0.56)**	**0.53 (0.50, 0.60)**
	SVM RBF	0.84 (0.78, 0.90)	0.39 (0.24, 0.49)	0.19 (0.18, 0.27)	**1.00 (0.86, 1.00)**	0.80 (0.71, 0.82)	0.29 (0.24, 0.33)	1.00 (0.98, 1.00)	0.43 (0.38, 0.49)	0.34 (0.28, 0.38)	0.64 (0.57, 0.71)	0.35 (0.28, 0.42)	0.44 (0.38, 0.52)

Values represent the median and interquartile range of 30 models independently developed by nested cross validation (see methods). Bolded values represent the best and/or most precise performance for each metric. Accuracy metrics, weighted averages, and correlation metrics are calculated at Youden’s *j* index. AUROC, area under the receiver-operator curve; AUPRC, area under the precision-recall curve; PPV, positive predictive value; NPV, negative predictive value; SVM, support vector machine; RBF, radial basis function; MCC, Matthew’s Correlation Coefficient.

All models were classified as highly or moderately accurate when predicting microbial confirmation from noninvasive specimen collection procedures (median AUROC 0.88, range: 0.83, 0.89; Fig 1, Table 3). Noninvasive model comparison demonstrated slightly more heterogeneity across modeling types: while the Bidirectional and SVM Linear models demonstrated the highest overall AUROCs (0.89), the Ridge model produced among the lowest overall misclassification error (0.11, IQR: 0.09, 0.18) and the highest specificity (0.89, IQR: 0.80, 0.93), PPV (0.28, IQR: 0.55, 0.35), NPV (0.98, IQR: 0.97, 0.99), F1 score (0.62, IQR: 0.48, 0.67), F2 score (0.54, IQR: 0.39, 0.59), Cohen’s Kappa (0.56, IQR: 0.40, 0.62), and MCC (0.59, IQR: 0.44, 0.66).

**Table 3:** Comparison of machine learning models to predict microbial confirmation using noninvasive sampling procedures.

Model Type		Performance		Accuracy Metrics at Youden’s *j* index					Weighted Averages			Correlation Metrics	
		AUROC	AUPRC	Misclass.	Sensitivity	Specificity	PPV	NPV	F1 Score	F2 Score	F0.5 Score	Cohen’s Kappa	MCC
Stepwise Regression	Forward Selection	0.88 (0.82, 0.90)	0.57 (0.43, 0.64)	0.17 (0.10, 0.27)	**0.88 (0.88, 1.00)**	0.81 (0.70, 0.90)	0.35 (0.26, 0.49)	**0.98 (0.98, 1.00)**	0.53 (0.41, 0.62)	0.41 (0.30, 0.54)	0.66 (0.60, 0.75)	0.44 (0.29, 0.57)	0.53 (0.39, 0.60)
	Backward Elimination	0.88 (0.82, 0.90)	0.57 (0.43, 0.64)	0.17 (0.10, 0.27)	**0.88 (0.88, 1.00)**	0.81 (0.70, 0.90)	0.35 (0.26, 0.49)	**0.98 (0.98, 1.00)**	0.53 (0.41, 0.62)	0.41 (0.30, 0.54)	0.66 (0.60, 0.75)	0.44 (0.29, 0.57)	0.53 (0.39, 0.60)
	Bidirectional	**0.89 (0.85, 0.94)**	0.61 (0.51, 0.69)	**0.11 (0.09, 0.18)**	**0.88 (0.88, 0.88)**	0.89 (0.81, 0.92)	0.47 (0.33, 0.54)	**0.98 (0.98, 0.99)**	0.61 (0.48, 0.67)	0.52 (0.38, 0.58)	0.74 (0.67, 0.76)	0.55 (0.39, 0.62)	**0.59 (0.46, 0.64)**
Regularization	LASSO	0.86 (0.84, 0.89)	0.56 (0.42, 0.66)	0.15 (0.10, 0.28)	**0.88 (0.88, 1.00)**	0.85 (0.69, 0.90)	0.39 (0.27, 0.50)	**0.98 (0.98, 1.00)**	0.54 (0.42, 0.62)	0.44 (0.32, 0.56)	0.69 (0.62, 0.72)	0.46 (0.31, 0.57)	0.53 (0.42, 0.59)
	Ridge	0.88 (0.85, 0.92)	0.65 (0.45, 0.67)	**0.11 (0.09, 0.18)**	0.88 (0.75, 0.88)	**0.89 (0.80, 0.93)**	**0.48 (0.35, 0.55)**	**0.98 (0.97, 0.99)**	**0.62 (0.48, 0.67)**	**0.54 (0.39, 0.59)**	0.70 (0.65, 0.78)	**0.56 (0.40, 0.62)**	**0.59 (0.44, 0.66)**
	Elastic Net	0.87 (0.83, 0.91)	0.48 (0.38, 0.63)	0.14 (0.10, 0.22)	**0.88 (0.88, 0.88)**	0.86 (0.79, 0.92)	0.42 (0.30, 0.50)	**0.98 (0.98, 0.99)**	0.56 (0.44, 0.64)	0.47 (0.34, 0.55)	0.70 (0.61, 0.76)	0.50 (0.32, 0.58)	0.54 (0.43, 0.61)
Decision Tree	Random Forest	0.87 (0.85, 0.91)	0.62 (0.45, 0.68)	0.15 (0.10, 0.22)	**0.88 (0.88, 1.00)**	0.84 (0.77, 0.90)	0.41 (0.29, 0.50)	**0.98 (0.98, 1.00)**	0.56 (0.44, 0.63)	0.46 (0.33, 0.55)	0.71 (0.65, 0.74)	0.49 (0.33, 0.57)	0.54 (0.43, 0.61)
	Gradient Boosted Tree	0.88 (0.85, 0.94)	**0.67 (0.53, 0.68)**	0.15 (0.11, 0.21)	**0.88 (0.88, 1.00)**	0.85 (0.79, 0.89)	0.41 (0.31, 0.47)	**0.99 (0.98, 1.00)**	0.57 (0.48, 0.61)	0.46 (0.36, 0.53)	0.72 (0.68, 0.77)	0.50 (0.38, 0.55)	0.56 (0.48, 0.60)
Support Vector Machines	SVM Linear	**0.89 (0.86, 0.94)**	0.65 (0.54, 0.76)	0.12 (0.04, 0.20)	0.88 (0.75, 0.88)	0.89 (0.78, 0.97)	0.45 (0.33, 0.76)	0.99 (0.97, 0.99)	0.58 (0.50, 0.80)	0.49 (0.38, 0.78)	0.71 (0.65, 0.84)	0.52 (0.41, 0.77)	0.55 (0.48, 0.77)
	SVM Polynomial	0.88 (0.85, 0.95)	0.60 (0.49, 0.69)	0.14 (0.09, 0.16)	**0.88 (0.88, 1.00)**	0.86 (0.82, 0.92)	0.42 (0.35, 0.54)	**0.98 (0.98, 1.00)**	0.57 (0.49, 0.66)	0.47 (0.40, 0.59)	**0.75 (0.65, 0.78)**	0.51 (0.41, 0.61)	0.57 (0.46, 0.64)
	SVM RBF	0.83 (0.77, 0.88)	0.42 (0.29, 0.57)	0.23 (0.18, 0.30)	0.88 (0.78, 0.88)	0.77 (0.68, 0.83)	0.29 (0.23, 0.35)	0.98 (0.97, 0.98)	0.41 (0.36, 0.50)	0.32 (0.27, 0.40)	0.59 (0.56, 0.68)	0.30 (0.25, 0.42)	0.39 (0.34, 0.48)

Values represent the median and interquartile range of 30 models independently developed by nested cross validation (see methods). Bolded values represent the best and/or most precise performance for each metric. Accuracy metrics, weighted averages, and correlation metrics are calculated at Youden’s *j* index. AUROC, area under the receiver-operator curve; AUPRC, area under the precision-recall curve; Misclass., misclassification error; PPV, positive predictive value; NPV, negative predictive value; SVM, support vector machine; RBF, radial basis function; MCC, Matthew’s Correlation Coefficient.

### Patient characteristics associated with microbial confirmation

**Fig 3:**
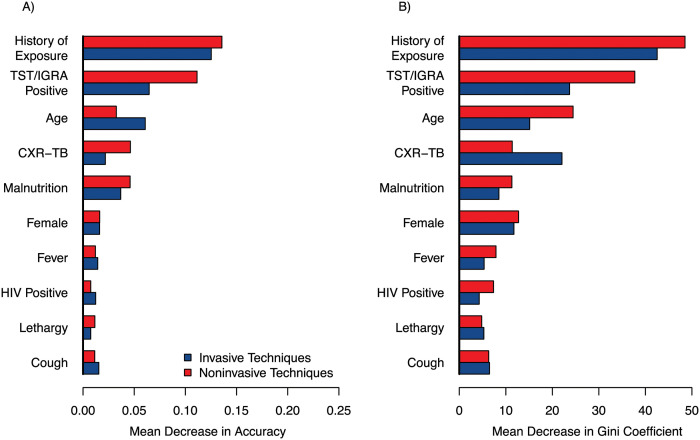
Comparison of parameter coefficient values ($\hat{\beta }$) for stepwise and regularization models. A) Microbial confirmation from invasive specimens only (GA and IS only); B) Microbial confirmation from noninvasive specimens only (NPA, stool, ST, FNA, urine, and blood only). WFA, weight-for-age; Hx. Exp., history of TB exposure; TST/IGRA+, positive for tuberculin skin test (TST) or interferon-gamma release assay (IGRA); CXR-TB, chest x-ray (CXR) consistent with tuberculosis disease.

**Fig 4:**
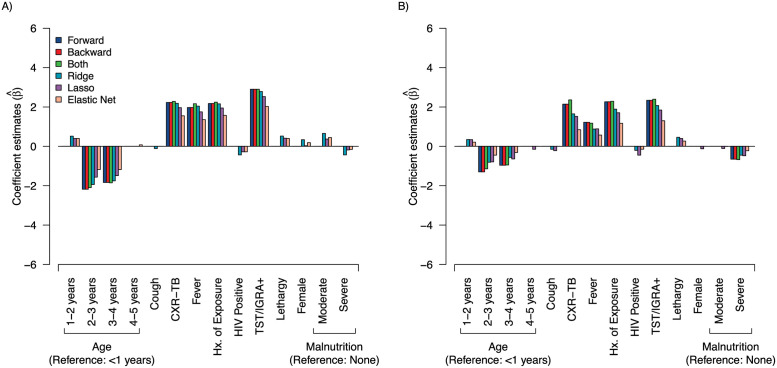
Factor influence in the Random Forest Model, by model. A) Mean decrease in accuracy, which expresses the accuracy lost by excluding a given variable. B) Mean decrease in Gini coefficient, which is a measure of how much each variable contributes to the homogeneity of the nodes and trees in the random forest model. For both measures, a higher value implies a higher importance in the Random Forest model. TST, tuberculin skin test; IGRA, interferon-gamma release assay; CXR-TB, chest x-ray (CXR) consistent with TB disease.

**Fig 5:**
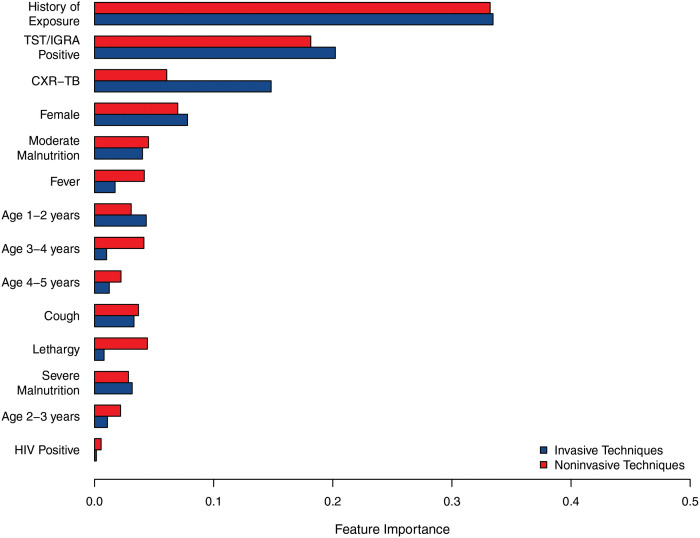
Feature importance in the Gradient Boosted Tree model. Feature importance is averaged across all of the decision trees within the model based on gain (the improvement of model accuracy attributed to branches containing the feature). TST, tuberculin skin test; IGRA, interferon-gamma release assay; CXR-TB, chest x-ray (CXR) consistent with TB disease.

**Fig 6:**
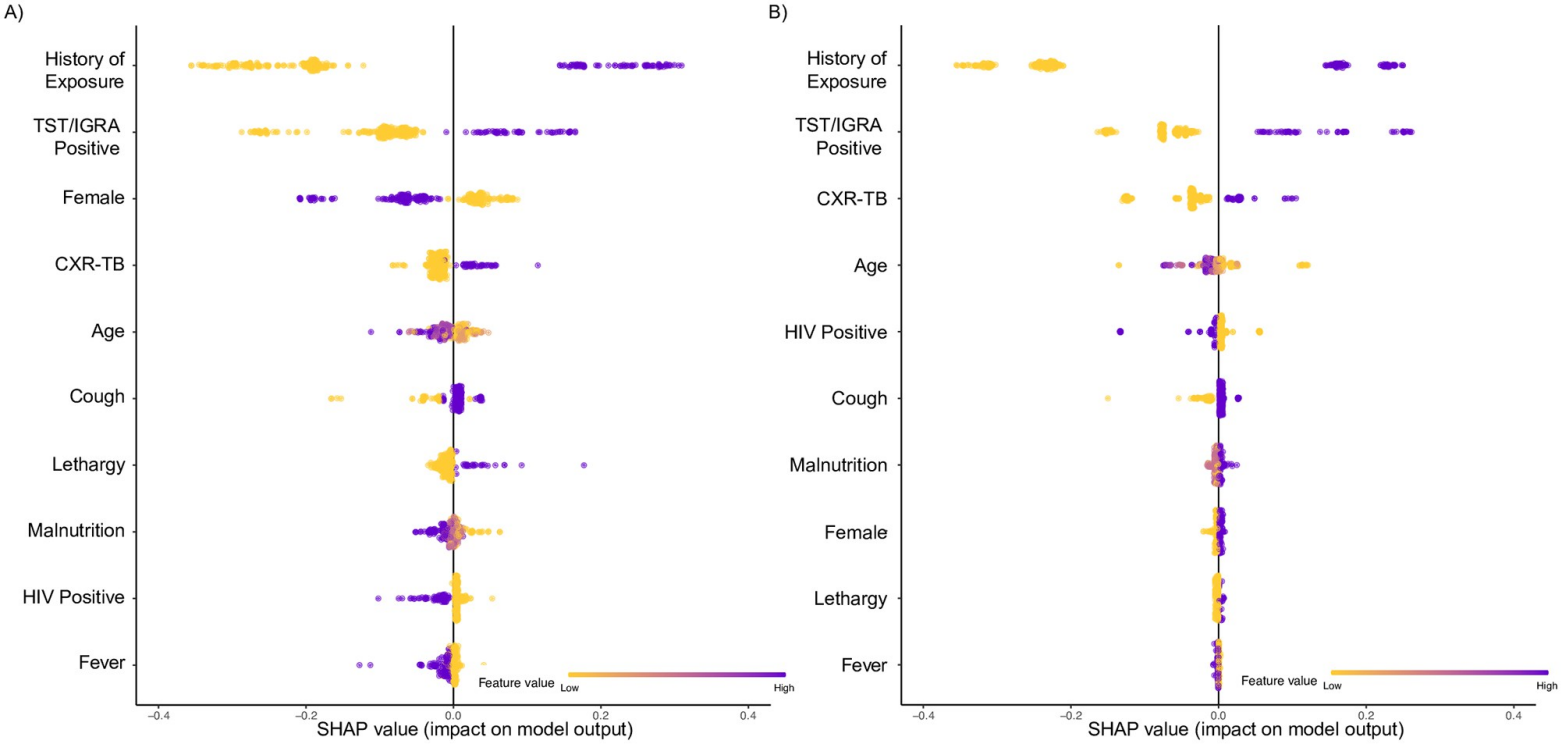
SHAP summary plot for SVM Polynomial Model. A) Invasive procedures; B) Non-invasive procedures. SHAP, Shapely Additive Explanations; SVM, Support Vector Machine; TST, tuberculin skin test; IGRA, interferon-gamma release assay; CXR-TB, chest x-ray (CXR) consistent with TB disease.

Differences in modeling approaches used in this analysis preclude a direct, objective comparison of features between models. However, clear patterns of influential predictors can be observed (Figs 3–6). History of TB exposure, immunological evidence of TB infection (positive TST or IGRA), CXR consistent with TB disease, were consistently influential factors across all models and outcomes. Importantly, clinical symptoms consistent with pediatric TB disease (cough, fever, malnutrition) seemed largely inconsequential across models and outcomes.

## Discussion

Applying machine learning methods to a large cohort of very young children with symptoms consistent with TB demonstrated that practical, easily obtainable clinical, demographic, and radiological information could be used to predict microbial confirmation of *M*. *tuberculosis* with a high degree of accuracy. These findings have two key implications: first, clinical teams seeking to determine if an invasive sampling procedure should be carried out for a child with presumptive TB could use such tools at the initial patient encounter for rapid decision-making. Knowledge that a child is very unlikely to produce a positive sample may reinforce a clinical TB diagnosis, thus hasten time to treatment initiation and improve patient outcomes. This is particularly useful in limited-resource, high incidence settings where patient follow up is challenging. Second, future research in pediatric TB, including vaccine trials and novel approaches of microbial confirmation among children, require confirmation using invasive sampling procedures as the reference standard. Researchers seeking to enroll a cohort of children with a high microbial yield can use these tools to guide enrollment criteria and flag screened participants with an increased likelihood of a positive result.

Several well-designed prediction models and diagnostic tools have been developed in children for clinical diagnoses of TB disease [24–27]. Clinical diagnoses are based on clinical and exposure history alone and are made in the absence of a microbially confirmed *M*. *tuberculosis* specimen, thus are considered unconfirmed TB cases [10]. Recently, Gunasekera *et al* [25] used predictors identified *a priori* in a logistic regression model to develop a treatment-decision algorithm for children with symptoms concerning for TB (AUROC of 0.75 when using clinical evidence only) and 0.87 when using clinical evidence plus CXR and Xpert MTB/RIF assay). Mier *et al* [28] used machine learning in a small number of pediatric TB patients (n = 59) to identify optimal antigen-biomarker combinations using whole blood analysis. Promisingly, the authors found several combinations of antigen-cytokine pairs that may improve future diagnostics over the current reference standard (interferon-gamma release assays; AUROCs 0.81–0.95). Brooks *et al* [26] used a classification and regression tree (CART) analysis to identify potential associations between covariates and incident TB in children under 14 years old. The authors found that immunological evidence of TB infection (positive TST) was strongly associated with incident TB. In addition, artificial intelligence has long been used to detect clinical TB in CXR readings with a high degree of accuracy (AUROCs: 0.92 to 0.99), however such use is almost exclusively focused on adult TB patients and their use in pediatric TB is limited [29,30].

Our analysis compliments these previous findings and is distinguished from this body of work in several important ways. First, in contrast to previous work seeking to improve decision-making of clinical diagnoses, to our knowledge this is the first analysis to use machine learning methods to predict laboratory-confirmed TB disease in young children. We further separate findings by the current invasive reference-standard procedures and secondary, noninvasive procedures commonly evaluated in diagnostic studies seeking to identify novel diagnostic tools. Second, our analysis intentionally used a small number of feasible and easily obtainable clinical, demographic, and radiologic patient-level factors and broadly defined categories to approximate real-word data collection practices in resource-limited settings. In contrast to examining a large number of complex predictors (i.e., blood biomarkers), our simplified approach improves the practical utilization of these tools in resource-limited settings that carries the largest burden of pediatric TB disease. Third, previous studies primarily use logistic regression with *a priori* defined predictors to examine covariates related to pediatric TB [27,31–33]. This analysis explores a wide range of machine learning classification approaches beyond standard logistic regression, such as decision trees and SVMs, to train models. Our results suggest that alternative modeling approaches, particularly SVMs, generally outperformed logistic regression and were more accurate in correctly predicting microbial confirmation. These findings may direct epidemiologic inquiry into alternative methodologies in the application of future clinical and diagnostic prediction models.

We estimated a range of accuracy and evaluation metrics to examine model performance. While taken together the metrics suggest that models performed well overall, we highlight that the best performing model had a PPV (precision) of 0.48, suggesting that around half of the children who were predicted to be microbially confirmed truly produced a positive sample. While this is markedly higher than both the bacteriologic yield of previous cohorts in similar settings of children with presumed TB disease (10%-15%) [7,8,34] and the expected yield of a random classifier in these data (11%), these results underscore a need for improved methods to predict microbial confirmation among children presumed to have TB disease.

We trained models using data from a well-described prospective cohort that implemented a meticulous and diverse array of sampling procedures to determine microbial confirmation of TB disease in young children. The results are based on rigorous machine learning analyses exploring a diverse range of modeling approaches and powered by a robust sample of pediatric patients with symptoms concerning for TB disease. However, this study has several important limitations. First, we intentionally coerced continuous variables, such as age and standardized WFA, into relatively broad categories and dichotomized other factors such as CXR readings (i.e., CXR consistent with TB vs. all other readings) and history of household TB exposure. In reality almost all factors exist on a spectrum and more accurate models may be designed to improve predictive ability for specific applications. Given that laboratory results are not always feasible in a resource limited setting, we used dichotomous HIV status as opposed to CD4 count, which is a more accurate indicator of immunological capacity. These data transformations also largely precluded the use of other popular machine learning methods, such as k-nearest neighbors (kNN) classification, as certain algorithms based on Euclidean distances perform better with continuous data and may have difficulty handling distance metrics from multiple dichotomous variables. Secondly, although we found similar factors upon qualitative examination of stepwise, regularization, and decision tree models, we could not objectively compare factors that may influence model development across modeling approaches. Moreover, determining factors that are influential in non-linear SVMs is not readily possible since data are transformed into another *j-*dimensional space that is incongruent with input space. We sought to address this by providing SHAP values to provide deeper insight into how the SVM models behaved (Fig 6). Third, despite being independently trained and subsequently tested on mutually exclusive datasets, both training and testing data represent the same source population, thus we are unable to assess the external generalizability of these models. Moreover, there may be unobserved factors that influence the results obtained in this analysis. As these data represent a single population, we cannot estimate the degree of influence of such factors using the empirical data. Model validation in external data representing diverse populations is the next logical and analytical step in refining these tools.

Applying a variety of machine learning approaches to data from a large cohort of children with suspected TB resulted in the identification of accurate and parsimonious prediction models of microbial confirmation. After extensive validation using data from other external populations, these data-driven findings may both facilitate clinical decision making and guide clinical research into novel biomarkers of TB infection among very young children. Future studies, particularly those in underrepresented and high-incidence settings, can extend the findings of this analysis and deepen our understanding of diagnostics in pediatric TB.
